# Metal-Free Dihydrogen Oxidation by a Borenium Cation: A Combined Electrochemical/Frustrated Lewis Pair Approach**

**DOI:** 10.1002/anie.201405721

**Published:** 2014-07-18

**Authors:** Elliot J Lawrence, Thomas J Herrington, Andrew E Ashley, Gregory G Wildgoose

**Keywords:** borenium cations, electrocatalysis, frustrated Lewis pairs, hydrogen, oxidation

## Abstract

In order to use H_2_ as a clean source of electricity, prohibitively rare and expensive precious metal electrocatalysts, such as Pt, are often used to overcome the large oxidative voltage required to convert H_2_ into 2 H^+^ and 2 e^−^. Herein, we report a metal-free approach to catalyze the oxidation of H_2_ by combining the ability of frustrated Lewis pairs (FLPs) to heterolytically cleave H_2_ with the in situ electrochemical oxidation of the resulting borohydride. The use of the NHC-stabilized borenium cation [(I*i*Pr_2_)(BC_8_H_14_)]^+^ (I*i*Pr_2_=C_3_H_2_(N*i*Pr)_2_, NHC=N-heterocyclic carbene) as the Lewis acidic component of the FLP is shown to decrease the voltage required for H_2_ oxidation by 910 mV at inexpensive carbon electrodes, a significant energy saving equivalent to 175.6 kJ mol^−1^. The NHC–borenium Lewis acid also offers improved catalyst recyclability and chemical stability compared to B(C_6_F_5_)_3_, the paradigm Lewis acid originally used to pioneer our combined electrochemical/frustrated Lewis pair approach.

In the global effort to develop carbon-neutral, alternative energy economies, H_2_ is often regarded as an attractive, clean fuel for the generation of electricity. However, in the absence of a suitable electrocatalyst, the electrochemical oxidation of H_2_ to generate 2 H^+^ and 2 e^−^ requires a large oxidative voltage, the energetic “driving force”, in order to overcome the sluggish kinetics of this electrochemical process.^1^ Conventional electrocatalyst materials typically use Pt or Pt-group metals (e.g. Pd, Ru, Rh) to significantly lower the voltage required for H_2_ oxidation reactions to occur. However, the high cost and relative scarcity of these precious metals pose a significant economic barrier to their widespread use in an H_2_-based energy economy.

Recently, we reported a new, metal-free approach to H_2_ oxidation that combines the ability of frustrated Lewis pairs (FLPs) to activate H_2_ with in situ electrochemistry, in order to convert H_2_ into 2 H^+^ and 2 e^−^ at cheap and abundant carbon electrodes.^2^ The chemical activation of H_2_ using FLPs was first introduced by Stephan and co-workers in 2006.^3^ They found that H_2_ may be heterolytically cleaved by a suitable combination of a sterically encumbered Lewis acid/base pair.^3^–^5^ Our initial approach involved the combination of the FLP activation of H_2_ using *t*Bu_3_P/B(C_6_F_5_)_3_ with the in situ electrochemical oxidation of the resulting [HB(C_6_F_5_)_3_]^−^ intermediate. This “combined electrochemical/frustrated Lewis pair approach” decreased the voltage required to oxidize H_2_ at a carbon electrode by 610 mV. However, a detailed mechanistic study of this particular system revealed several undesirable side reactions that prevented the electrocatalytic system from turning over.^2^

Herein, we extend our “combined electrochemical/FLP approach” beyond simple arylborane Lewis acid catalysts, to the NHC-stabilized borenium cation [(I*i*Pr_2_)(BC_8_H_14_)]^+^, (**1**^+^ in Scheme 1; I*i*Pr_2_=C_3_H_2_(N*i*Pr)_2_, NHC=N-heterocyclic carbene). The ability of **1**^+^ to activate H_2_, when combined with Lewis base *t*Bu_3_P, has been reported previously, and the lack of electron-withdrawing groups in **1**^+^ imparts considerable hydridic character to the neutral NHC–borane adducts, even in comparison with anionic borohydrides such as [HB(C_6_F_5_)_3_]^−^.^6^, ^7^ Indeed, B(C_6_F_5_)_3_ is capable of abstracting H^−^ from **1**-H to give the salt **1**[HB(C_6_F_5_)_3_].^6^ In addition, the bond-dissociation energy of the B—H bond is weakened by the coordination of a carbene ligand.^8^, ^9^ As we demonstrate herein, the combination of these properties improves the chemical stability of borenium Lewis acid catalysts toward unwanted side reactions (e.g. reaction with the solvent) and further decrease the voltage required to oxidize H_2_ at a carbon electrode by 910 mV (equivalent to 175.6 kJ mol^−1^).

**1 Scheme:**
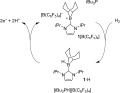
Electrooxidation of the H_2_-activated *t*Bu_3_P/**1**[B(C_6_F_5_)_4_] frustrated Lewis pair (FLP) results in the electrochemical generation of two protons and two electrons.

We prepared **1**-H according to the method of Farrell et al.^6^, and its redox properties were explored using cyclic voltammetry. A single oxidation wave was observed at +(0.58±0.01) V versus Cp_2_Fe^0/+^ (Figure 1) with no corresponding reduction wave at scan rates up to 5 V s^−1^. The observed voltammetric behavior of **1**-H is very similar to that of [HB(C_6_F_5_)_3_]^−[2]^ (Figure 1 b) and therefore we propose the mechanism shown in Scheme 2 to account for the observed voltammetry: upon the application of an oxidizing potential, **1**-H undergoes a one-electron oxidation to form a transient [**1**-H]^.+^ species. This then undergoes rapid dissociation in solution to give H^+^ and a neutral radical, **1**. As the applied potential is very positive compared to the formal potential for the **1**^+^/**1**^.^ couple (see below), this radical undergoes a second one-electron oxidation to generate **1**^+^. This second oxidation occurs in competition with side reactions: 1) the decomposition of **1**^.^ by the solvent, and 2) the reaction between electrogenerated H^+^ and a second incoming molecule of **1**-H to regenerate **1**^+^ and H_2_ (see below).

**Figure 1:**
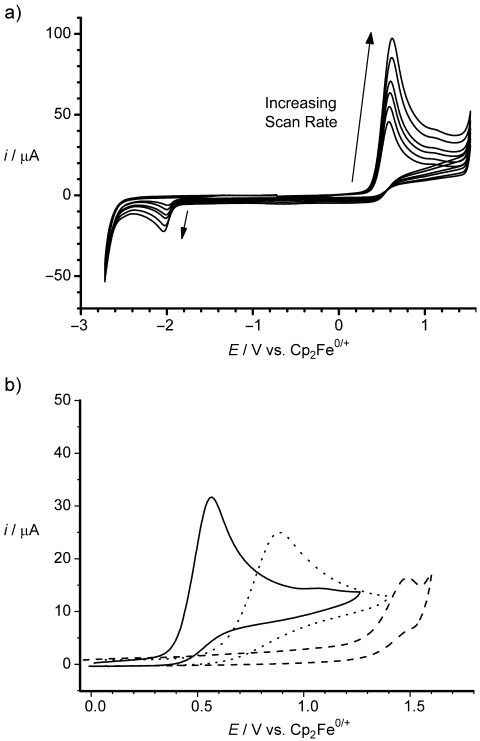
a) Overlaid cyclic voltammograms showing the complete potential window for **1**-H (2.0 mm, CH_2_Cl_2_) over the voltage scan-rate range 200–1000 mV s^−1^. b) Cyclic voltammograms comparing the oxidation potentials of **1**-H (solid line, 2.0 mm), [HB(C_6_F_5_)_3_]^−^ (dotted line, 2.0 mm), H_2_ (dashed line, 2.0 mm) in CH_2_Cl_2_ at voltage scan rates of 100 mV s^−1^.

**Scheme 2:**
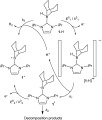
Proposed mechanism and associated thermodynamic and kinetic parameters used in the digital simulation of the voltammetric oxidation of **1**-H at a GCE.

On the reverse scan (Figure 1 a), a smaller, irreversible reduction wave was observed at −(1.97±0.01) V versus Cp_2_Fe^0/+^. This corresponds to the one-electron reduction of electrogenerated **1**^+^, and was confirmed independently by performing cyclic voltammetry on an authentic sample of [**1**][B(C_6_F_5_)_4_] (see the Supporting Information, Figure S1. It is worth noting that the reduction of **1**^+^ occurs at a voltage that is 150 mV more negative than that of the archetypal Lewis acid B(C_6_F_5_)_3_.^10^ This result suggests that **1**^+^ is significantly less electrophilic than B(C_6_F_5_)_3_ and accounts for the increased hydridic character (and lower oxidation potential) of **1**-H compared to [HB(C_6_F_5_)_3_]^−^. The reduction of **1**^+^ to form **1**^.^ is irreversible at all scan rates up to 5 V s^−1^. This is due to the rapid decomposition of **1**^.^ in solution to form a mixture of various redox-inactive, four-coordinate borates. This process presumably occurs in an analogous manner to the solvent-specific decomposition of the boron-centered radical anion, [B(C_6_F_5_)_3_]^.−^,^10^ as one would expect the unpaired electron in **1**^.^ to be localized in a B—C π-bonding orbital with notable polarization toward the boron atom, similar to the previously reported NHC–boryl radical, [(C_3_H_2_(NMe)_2_)BMes_2_]^.^.^11^–^13^ However, the reduction wave (and hence the concentration) of electrogenerated **1**^+^, arising from the oxidation of **1**-H, is much larger than that observed in the B(C_6_F_5_)_3_/[HB(C_6_F_5_)_3_]^−^ system.^2^

Treatment of **1**-H with one equivalent of Jutzi’s strong acid, [H(OEt_2_)_2_][B(C_6_F_5_)_4_],^14^ resulted in the quantitative conversion of **1**-H to **1**[B(C_6_F_5_)_4_]. This observation confirms the protolytic side-reaction between electrogenerated H^+^ and **1**-H. The result is in stark contrast to the corresponding reaction of [HB(C_6_F_5_)_3_]^−^ with H^+^, in which neither the free Lewis acid, B(C_6_F_5_)_3_, nor the etherate adduct, Et_2_O**⋅**B(C_6_F_5_)_3_, were present.^2^ This result, together with the observation of significant amounts of electrogenerated **1**^+^ in the voltammetry of **1**-H, suggests that Lewis acid **1**^+^ has a markedly improved stability toward H^+^ compared to B(C_6_F_5_)_3_.

Digital simulation of the cyclic voltammetric data for the oxidation of **1**-H was undertaken in order to extract pertinent mechanistic, thermodynamic, and kinetic parameters (see the Supporting Information for further details). The experimental and simulated cyclic voltammograms were in very good agreement (Figure 2) when the simulation was performed in accordance with the mechanism proposed in Scheme 2.

**Figure 2:**
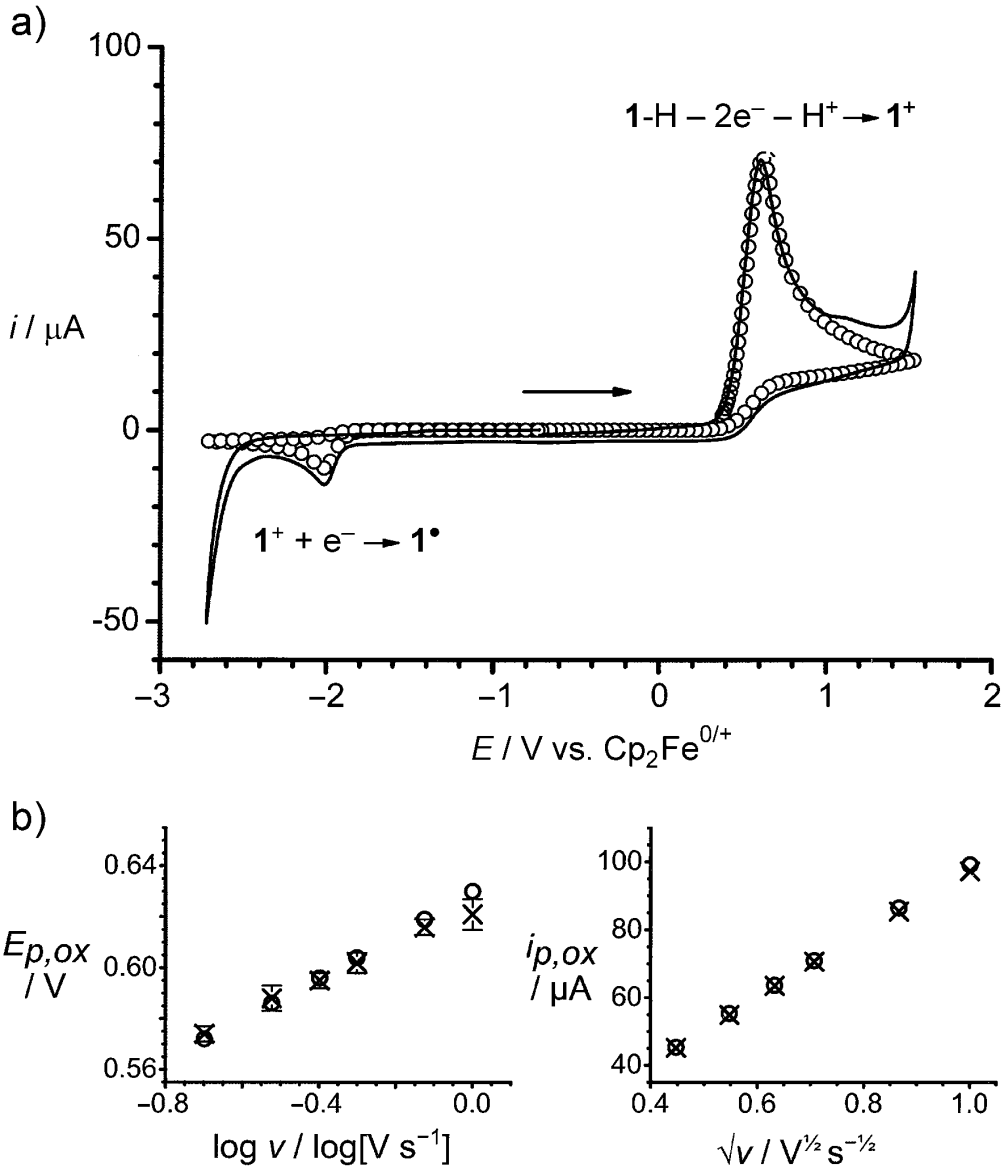
a) Simulated (circles) and experimental (line) cyclic voltammograms showing the full potential window for **1**-H (2.0 mm, CH_2_Cl_2_) at a voltage scan rate (*ν*) of 500 mV s^−1^. Comparisons between experimental and simulated data showing the quality of fitting for: b) the oxidative peak potential (*E*_p, ox_) versus the logarithm of voltage scan rate (*v*), and c) the oxidative peak current (*i*_p,ox_) versus the square root of voltage scan rate (*v*).

By comparing the results of our digital simulations for the **1**-H/**1**^+^ system to the [HB(C_6_F_5_)_3_]^−^/B(C_6_F_5_)_3_ system reported previously,^2^ three observations are apparent: 1) the rate of oxidation of **1**-H is around 40 times greater than that of [HB(C_6_F_5_)_3_]^−^, leading to larger oxidative currents (see Figure 1 b); 2) the oxidation of **1**-H occurs at a potential that is around 300 mV lower than for [HB(C_6_F_5_)_3_]^−^, corresponding to a significantly large net decrease in the potential required to oxidize H_2_ at a glassy carbon electrode of 910 mV (see Figure 1 b); 3) the rate of unwanted protonation of **1**-H by electrogenerated H^+^ to generate **1**^+^ and H_2_ (*k*_3_) is 3×10^5^ times slower than in the [HB(C_6_F_5_)_3_]^−^ system. Indeed, a simulation reveals that only 30–40 % of **1**^+^ that is reduced at the electrode surface is generated through this undesirable protolytic H_2_ regeneration process, with the majority of **1**^+^ being generated through the two-electron oxidation of **1**-H. This result, combined with the greater chemical stability of **1**^+^ toward unsolvated H^+^, demonstrates a significant improvement over the previous [HB(C_6_F_5_)_3_]^−^/B(C_6_F_5_)_3_ system.^2^

Encouraged by these findings, we next investigated the application of **1**^+^ in the in situ H_2_ oxidation using a combined electrochemical/FLP approach and, importantly, whether this system could be cycled subsequently. H_2_ was admitted to a 1:1 solution of [**1**][B(C_6_F_5_)_4_]/*t*Bu_3_P in CH_2_Cl_2_ containing [*n*Bu_4_N][B(C_6_F_5_)_4_] electrolyte. The reaction mixture was stirred in a sealed flask overnight to allow H_2_ cleavage by the FLP to go to completion. The resulting solution, containing **1**-H (as identified from an initial cyclic voltammogram of the solution) and [*t*Bu_3_PH][B(C_6_F_5_)_4_], was then subjected to bulk electrolysis, holding the electrode at the oxidation potential of **1**-H until complete conversion was achieved. Afterward, an aliquot of *t*Bu_3_P (equimolar to the original quantity of [**1**][B(C_6_F_5_)_4_]) was added to the solution, and the reaction mixture was again sealed under H_2_ atmosphere overnight. This cycle of bulk electrolysis and subsequent “recharging” under H_2_ atmosphere was repeated a total of three times (see the Supporting Information, Figure S2). The total charge that was passed (equivalent to the concentration of **1**-H) in the second cycle of electrolysis was 75 % of that of the first cycle, clearly indicating that the system is capable of turning over more than a single cycle. However, upon the third cycle of electrolysis, the current and charge passed had dropped to below 5 % of the initial cycle. Closer investigation revealed that the capacitive charging current had diminished considerably and cyclic voltammetry of the solution provided evidence of electrode fouling as a result of the presence of a broad, ill-defined, surface-bound oxidation wave over the range 0.2–0.8 V versus Cp_2_Fe^0/+^. This behavior most likely results from oxidative polymerization of the phosphine Lewis base, which, assuming that 100 % of **1**-H could be regenerated in each step, was subsequently present in excess. Although the oxidation potential of *t*Bu_3_P occurs at slightly more positive potentials than that of **1**-H, the oxidation of free *t*Bu_3_P may still occur to an extent. While it is not yet clear whether improved turnover of the system may have been possible in the absence of fouling, the fact that **1**-H can be recycled even once is an improvement over the initial [HB(C_6_F_5_)_3_]^−^/B(C_6_F_5_)_3_ system, in which all previous recycling attempts failed. Note that while our system is electrocatalytic in the Lewis acid component, it is stoichiometric in the Lewis base *t*Bu_3_P, as this study is only concerned with the anodic half-cell reaction, that is, the H_2_ oxidation. If our system were to be utilized as part of a complete electrochemical cell reaction (e.g. a fuel cell), then clearly it would require coupling to a suitable cathodic half-cell reaction, which would be capable of consuming the protons generated and thus of closing the catalytic cycle.

In conclusion, we successfully demonstrated the use of an NHC-stabilized borenium cation, **1**^+^, in the electrocatalytic oxidation of H_2_ using a combined electrochemical/frustrated Lewis pair approach. Using borenium cations as Lewis acid components of the FLP has several advantages over the initial B(C_6_F_5_)_3_-based system reported previously:^2^ the oxidative voltage (driving energy) required to oxidize H_2_ at a carbon electrode is significantly decreased by almost one Volt (910 mV, ca. 175.6 kJ mol^−1^); the rate of H_2_ oxidation is faster, thus producing larger currents; and the NHC-stabilized borenium Lewis acid catalyst is more resistant to unwanted protolytic side-reactions. These factors combine to make the NHC-stabilized borenium system somewhat “rechargeable”, allowing the in situ regeneration of **1**-H from **1**^+^ in the presence of *t*Bu_3_P and H_2_, with 75 % efficiency after the first cycle.

However, our approach is still in its infancy and several challenges remain. The rate of H_2_ cleavage by the FLP remains the rate-determining step, and the neutral radical intermediate, **1**^.^, is still susceptible to decomposition in the solvent. Both of these factors may limit the “turnover” or recycling of this system, which would render it truly electrocatalytic. However, the advantage of FLP systems is that they are inherently “tunable” with new, improved combinations of Lewis acids and bases reported apace. The structural and electronic factors that affect the ability of a variety of different synthetically accessible borenium cations to effect the FLP activation of H_2_ is complex,^15^, ^16^ and is still being developed. Thus, the incorporation of other borenium Lewis acid catalysts that may proffer improved electrochemical/frustrated Lewis pair systems remains to be explored.
