# Supplementary material for: Metal-Free Dihydrogen Oxidation by a Borenium Cation: A Combined Electrochemical/Frustrated Lewis Pair Approach
Source: Angew Chem Int Ed Engl. 2014 Jul 18;53(37):9922–5. doi: 10.1002/anie.201405721 (PMC4257500; doi:10.1002/anie.201405721)
Supplement: Supplementary file 1 [file anie0053-9922-SD1.pdf]

Angewandte Chemie
Eine Zeitschrift der Gesellschaft Deutscher Chemiker

Supporting Information

© Wiley-VCH 2014

69451 Weinheim, Germany

Metal-Free Dihydrogen Oxidation by a Borenium Cation: A Combined Electrochemical/Frustrated Lewis Pair Approach**

Elliot J. Lawrence, Thomas J. Herrington, Andrew E. Ashley, and Gregory G. Wildgoose**

anie_201405721_sm_miscellaneous_information.pdf

Supporting Information

Experimental details S2

Electrochemistry S3-S5

Experimental Details

General methods and materials

All synthetic reactions and manipulations were performed under a dry N_2 atmosphere using standard Schlenk-line techniques on a dual manifold vacuum/inert gas line or either an MBraun glovebox. Anhydrous solvents were dried via distillation over appropriate drying agents, sparged with N_2 gas to remove any trace of dissolved O_2 and stored in ampules over activated 4 Å molecular sieves. Deuterated NMR solvents ($CDCl_3$, 99.8 %) were purchased from Cambridge Isotope Laboratories Inc. and were dried over P_4O_{10}, degassed using a triple freeze-pump-thaw cycle and stored over activated 4 Å molecular sieves. **1-H**,[1] **1**[$B(C_6F_5)_4$],[1] [^{n}Bu_4N][$B(C_6F_5)_4$][2–4], [$H(OEt_2)_2$][$B(C_6F_5)_4$][5], and ^{t}Bu_3P[6] were prepared according to literature methods. NMR spectra were recorded using either a Bruker Avance DPX-300 or DPX-500 MHz spectrometer. Chemical shifts are reported in ppm and are referenced relative to appropriate standards.

Proton-mediated generation of [1][$B(C_6F_5)_4$] from 1-H

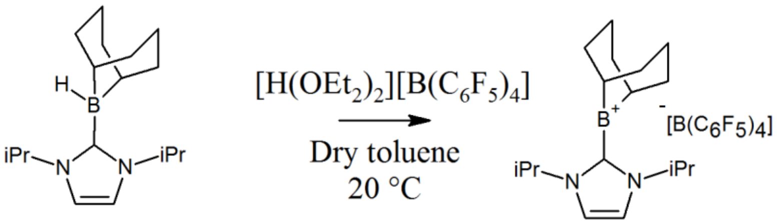

To a solution of **1**-H (0.14 g, 0.53 mmol) in toluene (5 mL) was added a suspension of [$H(OEt_2)_2$][$B(C_6F_5)_4$] (0.41 g, 0.53 mmol) in toluene (10 mL). Vigorous effervescence was immediately observed and a gelatinous precipitate was formed. The reaction mixture was left to stir overnight before it was concentrated *in vacuo* to give a white powder. The residue was recrystallized from chlorobenzene (3 mL) overnight at −20 °C. The crystals were then filtered and washed with dry petrol (4 × 1 mL). The crystals were dried in vacuo to yield **1**[$B(C_6F_5)_4$] (0.42 g, 0.41 mmol, 77 %) as a white powder. ^{1}H NMR (300 MHz, $CDCl_3$): δ= 7.61 (s, 2H), 4.77 (m, ^{3}J(H,H)=6.8 Hz, 2H; CH), 2.44-2.24 (br. m, 6H), 2.20-2.12 (br. m, 2H) 2.10-1.94 (br. m, 4H), 1.88-1.59(br. m, 2H), 1.68 (d, ^{3}J(H,H)=6.7 Hz, 12H; CH_3); ^{19}F NMR (471 MHz, $CDCl_3$): δ= −137.7 (m, 8F; *ortho*-F), −168.2 (m, 4F; *para*-F), −172.1 (m, 8F; *meta*-F); ^{11}B NMR (160 MHz, $CDCl_3$): δ= 83.8 (br), −16.7 (s).

References

[1] J. M. Farrell, J. A. Hatnean, D. W. Stephan, *J. Am. Chem. Soc.* **2012**, *134*, 15728–15731.

[2] E. Martin, D. L. Hughes, S. J. Lancaster, *Inorg. Chim. Acta* **2010**, *363*, 275–278.

[3] E. J. Lawrence, V. S. Oganesyan, G. G. Wildgoose, A. E. Ashley, *Dalton Trans.* **2013**, *42*, 782–789.

[4] R. J. LeSuer, C. Buttolph, W. E. Geiger, *Anal. Chem.* **2004**, *76*, 6395–6401.

[5] P. Jutzi, C. Müller, A. Stammler, H.-G. Stammler, *Organometallics* **2000**, *19*, 1442–1444.

[6] R. C. Srivastava, *J. Chem. Res. (S)* **1985**, 330–331.

Electrochemistry

General methods

All electrochemical experiments were performed using a PGSTAT 302N computer-controlled potentiostat. Cyclic voltammetry (CV) was performed using a three-electrode configuration consisting of either a glassy carbon macrodisk working electrode (GCE) (diameter of 3 mm; BASi, Indiana, USA) combined with a Pt wire counter electrode (99.99 %; GoodFellow, Cambridge, UK) and a Ag wire pseudoreference electrode (99.99 %; GoodFellow, Cambridge, UK). The GCE was polished between experiments using successive grades of diamond paste slurries from 3.0 to 0.1 µm (Kemet, Maidstone, UK), and then briefly sonicated and rinsed in ethanol to remove any adhered microparticles. The metal electrodes were then dried in an oven at 100 °C to remove any residual traces of water, the GCE was left to air dry and residual traces of water were removed by applying a vacuum to the glovebox port. The electroactive area for each experiment was calibrated using a 5 mM ferrocene solution in CH_3CN, containing 0.1 M [$^nBu_4N$][PF_6] as the supporting electrolyte. The electroactive area was accurately determined by construction of a Randles-Sevcik plot from cyclic voltammograms recorded at varying scan rates (50-750 mVs^{-1}).[1] The Ag wire pseudoreference electrodes were calibrated to the ferrocene/ferrocenium couple in CH_2Cl_2 at the end of each run to allow for any drift in potential, following IUPAC recommendations.[2] Controlled potential bulk electrolyses were performed using a three-electrode configuration consisting of a carbon felt (99.0 %; Alfa Aesar, Massachusetts, USA) working electrode combined with a Ag wire pseudo-reference electrode (99.99 %, GoodFellow, Cambridge, UK) and a Pt gauze counter electrode (52 mesh woven from 0.1 mm diameter wire, 99.9 %; Alfa Aesar, Massachusetts, USA). All electrochemical measurements were performed at ambient temperatures under an inert N_2 atmosphere in CH_2Cl_2 containing 0.05-0.10 M [$^nBu_4N$][$B(C_6F_5)_4$] as the supporting electrolyte. All measurements were iR-compensated to within 80 ± 5 % of the solution uncompensated resistance.

Digital simulation

CV simulations were performed on baseline-corrected data using DigiElch – Professional (v 7.030) software. The postulated mechanism in Scheme 2 was applied to the baseline-corrected and iR-compensated cyclic voltammograms of **1**-H, taken over a scan rate range of 200-1000 mV s^{-1}. Any non-Faradaic current, resulting from double-layer capacitance, was not accounted for in the simulations. The diffusion coefficients for **1**-H and $\mathbf{1}^+$ were found to be 1.28 and 0.99 × 10^{-5} cm^2 s^{-1}, respectively, from diffusion-ordered NMR spectra (DOSY) in CD_2Cl_2. The diffusion coefficient values were left fixed for all electrochemical simulations. The electrochemical parameters for the reduction of electrogenerated $\mathbf{1}^+$ were determined by separate fits to the voltammetric data obtained for the reduction of **1**[$B(C_6F_5)_4$] with an EC mechanism over the same range of voltage scan rates. The experimental and simulated cyclic voltammograms were found to be in excellent agreement (Figure 2) when simulated according to the proposed mechanism in Scheme 2. The heterogeneous electrochemical parameters: formal potential, E^0_f, standard electron transfer rate constant, k^0, and charge transfer coefficient, α, are listed in SI Table 1 , together with the rate constants, k_1 -k_3, for the associated homogeneous chemical follow-up reactions.

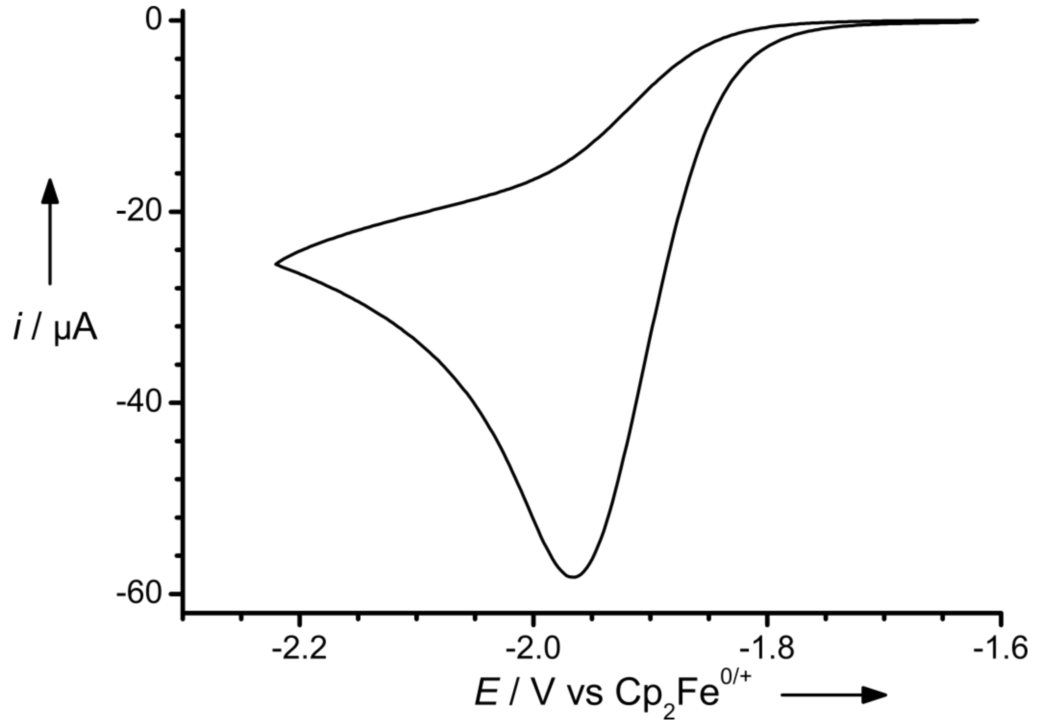

SI Figure 1. Cyclic voltammogram showing the reduction of authentic [**1**][$B(C_6F_5)_4$] (4.3 mM, CH_2Cl_2) at a voltage scan rate of 100 mV s^{-1}.

SI Table 1. Globally optimized best-fit thermodynamic and kinetic parameters for the oxidation of **1**-H at a GCE, obtained from digital simulation of the voltammetric data following the proposed mechanism in Scheme 2.

Redox process	E^0 / V vs $Cp_2Fe^{0/+}$	k^0 / cm s^{-1}	α
**1**-H ⇌ **1**-$H^{\bullet+}$ + e^-	+0.736 ± 0.01	4.0 ± 0.1 × 10^{-1}	0.5
**1**$^{\bullet}$ ⇌ **1**$^{+}$ + e^-	−1.935 ± 0.01	3.4 ± 0. 1 × 10^{-3}	0.6
**Chemical step**	**Rate constant, *k***		
**1**-$H^{\bullet+}$ → **1**$^{\bullet}$ + H^+	$k_1 > 3.0 \times 10^{11}$ $M^{-1}s^{-1}$		
**1**$^{\bullet}$ → Decomposition products	$k_2 > 2 \times 10^{13}$ s^{-1}		
**1**-H + H^+ → **1**$^{+}$ + H_2	$k_3 = 50$ s^{-1}		

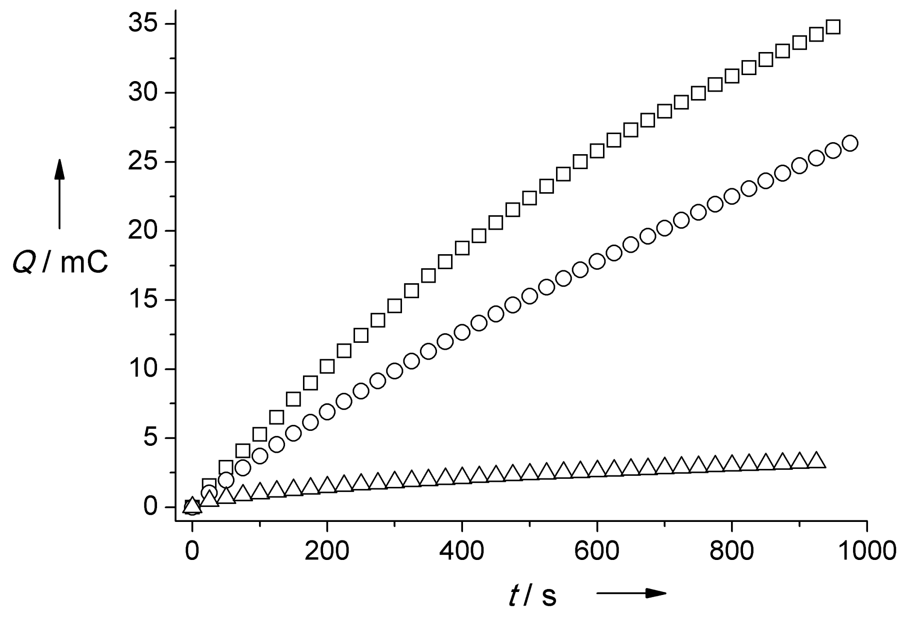

SI Figure 2. Repeat bulk electrolysis cycles of **1**-H, generated from the overnight exposure of a 5 mM **1**[B(C_6F_5)$_4$]/^{t}Bu$_3$P solution (containing supporting electrolyte) to H_2, demonstrate that the system is somewhat recyclable (cycle 1, square; cycle 2, circle; cycle 3, triangle).

References

[1] R. G. Compton, C. E. Banks, *Understanding Voltammetry*, Imperial College Press, **2011**.

[2] G. Gritzner, J. Kůta, *Electrochim. Acta* **1984**, *29*, 869–873.
